# Speech-Language Pathologists’ Support for Parents of Young d/Deaf Multilingual Learners

**DOI:** 10.1093/deafed/enac024

**Published:** 2022-08-20

**Authors:** Pauline van der Straten Waillet, Cécile Colin, Kathryn Crowe, Brigitte Charlier

**Affiliations:** Centre Comprendre et Parler, Bruxelles 1200, Belgium; Laboratoire Cognition Langage & Développement (LCLD), Centre de Recherche en Cognition et Neurosciences (CRCN), Université Libre de Bruxelles, Bruxelles 1050, Belgium; Laboratoire Cognition Langage & Développement (LCLD), Centre de Recherche en Cognition et Neurosciences (CRCN), Université Libre de Bruxelles, Bruxelles 1050, Belgium; School of Health Sciences, University of Iceland, Reykjavík 102, Iceland; School of Education, Charles Sturt University, Bathurst, NSW 2795, Australia; Centre Comprendre et Parler, Bruxelles 1200, Belgium; Laboratoire Cognition Langage & Développement (LCLD), Centre de Recherche en Cognition et Neurosciences (CRCN), Université Libre de Bruxelles, Bruxelles 1050, Belgium

## Abstract

Increasing cultural and linguistic diversity among children and families brings new challenges for early intervention professionals. The purpose of this study was to identify the specific roles and needs of speech-language pathologists (SLPs) who practice in early intervention settings with culturally and linguistically diverse families of d/Deaf multilingual learners (DMLs). Thirteen SLPs completed an online survey about their practices and needs. Interviews were conducted with five parents of DMLs. Results showed that SLPs have lower self-satisfaction with families of DMLs compared to mainstream families. Parents were highly satisfied with the support they received. Both groups of participants reported a need for specific tools or adaptations, especially if there was no shared language. Thematic analysis identified three themes: communication and partnership, professional resources for responding to diversity, and diversity of parental profiles. This article provides an insight into the perspectives of both professionals and culturally and linguistically diverse parents, and identifies specific aspects of early intervention services with parents of DMLs: developing partnership in the context of cultural and/or linguistic differences, discussing topics related to multilingualism, and providing highly adaptable family-centered services.

Cultural and linguistic diversity is increasing in many regions around the world. In nations traditionally considered to be monolingual English societies, such as the United States and Australia, 20% of school-aged children are reported to speak a language other than English at home (Australian Bureau of Statistics, 2017; Federal Interagency Forum on Child and Family Statistics, 2019). In a survey conducted in six European cities (Extra & Yağmur, 2011), the proportion of school-aged children speaking a home language different to the societal language has been reported to range from 10% (Madrid, Spain) to 80% (Brussels, Belgium). In each of these cities, between 50 and 90 different languages were reported to be used. For professionals who work with young children with communication difficulties and their families, addressing this linguistic diversity is increasingly becoming a part of daily practice rather than an exceptional occurrence (Bijleveld, Estienne, & Vander Linden, 2014; Cycyk, De Anda, Moore, & Huerta, 2021; Newbury, Poole, & Theys, 2020). This level of linguistic diversity challenges professionals’ ability to offer enriching and effective services to families, especially when language and communication are at the very core of a professional’s work, as it is for speech-language pathologists (SLPs). Due to this, SLPs may have specific roles and needs in supporting culturally and linguistically diverse families in early intervention (EI).

These changes in linguistic diversity in the general community are, unsurprisingly, paralleled in the number of children with hearing loss who are growing up in multilingual spoken language contexts (Leigh & Crowe, 2015; Rhoades, Price, & Perigoe, 2004; van der Zee & Dirks, 2022). Such children are referred to as d/Deaf multilingual learners (DMLs), and may be exposed to and/or acquiring two or more spoken languages (Crowe & Guiberson, 2019). Early intervention is essential for optimal outcomes for children with hearing loss, and therefore many DMLs and their families participate in early intervention, particularly Family-Centered Early Intervention (FCEI; van der Zee & Dirks, 2022). However, professionals working with multilingual children and families (with and without hearing loss) have often reported barriers to working effectively with multilingual clients (Crowe & Guiberson, 2021). There has been little research to describe the experience of being the parent of a multilingual child involved in FCEI. In addition, the existing literature is principally reports on contexts where the dominant language of the community is English. The investigation reported in this paper contributes to the existing literature by examining FCEI with DMLs from the viewpoint of professionals (SLPs) and parents in a context where the dominant community language is French.

## The Basics of Early Intervention

EI is defined as the process of providing services and supports to infants, toddlers, and their families when a child has, or is at risk of, a developmental delay, disability, or health condition that may affect typical development and learning (American Speech-Language-Hearing Association (ASHA), 2022). For children who have, or are at risk of, speech or language difficulties, the main goal of EI services is to enrich the child’s experiences so as to increase the child’s opportunity to receive, and respond to, appropriate language in meaningful social interactions (Wing et al., 2007). Because children develop their language through the opportunities provided by everyday parent–child interactions (Ford et al., 2020; Kuhl et al., 2008), many evidence-based EI programs include parent training that targets parent–child interactions. For example, the Hanen Program (Girolametto & Weitzman, 2006), Enhanced Milieu Teaching (Hemmeter & Kaiser, 1994), and shared book reading interventions (Noble et al., 2019).

For children with hearing loss and DMLs, best practice for EI implements a *family-centered early intervention* (FCEI) approach (Cannon & Guardino, 2022; Moeller, Carr, Seaver, Stredler-Brown, & Holzinger, 2013). FCEI is based on six elements: family as the unit of attention, family choice, family strengths, family–professional relationship, family needs, and individualized family services (Allen & Petr, 1996). This intervention model considers the diversity of families (i.e., culture, economic status, work, religion, etc.) and the use of culturally sensitive practices (Voss & Stredler-Brown, 2017). In FCEI, each family’s values, goals, and aspirations need to be clarified so that the intervention process be individualized to respect the family’s unique needs, preferences, and context (Moeller et al., 2013). FCEI places the family at the center of the EI process and focuses on empowering parents in informed decision-making, advocacy, and being the primary intervention providers for their child. This is particularly important for the families of children with hearing loss, as hearing loss can be identified in the first days of life and intervention to support the child’s communication begins as soon as possible after this (Stewart, Slattery, & McKee, 2021). Evidence shows that use of an FCEI approach can have positive impacts on parental self-efficacy, decision making, family satisfaction, and empowerment (Crais, 1991; Dirks & Szarkowski, 2022; Dunst, Trivette, & Hamby, 2007; Pighini, Goelman, Buchanan, Schonert-Reichl, & Brynelsen, 2014). A FCEI approach also highlights the importance of families’ preferences, one of three components of evidence-based practice (Dollaghan, 2007; Roulstone, 2011).

## Early Intervention with Culturally and Linguistically Diverse Families

To provide EI services to culturally and linguistically diverse (CLD) families, certain conditions are required on the part of the professionals (e.g., educators, SLPs), on the part of the parents, and between these two partners. First, professionals have a responsibility to know about typical multilingual development, be able to differentiate speech and language differences from disorders in multilingual populations, and to debunk myths associated with language development and use in CLD populations (Guiberson, 2013). It is recommended that professionals foster partnership and provide guidance to families to promote supportive communicative environments for children in all their languages (International Expert Panel on Multilingual Children’s Speech, 2012; Moeller et al., 2013; Ruiz et al., 2006). Professionals also need to be able to provide culturally and linguistically responsive services to children and families in EI settings (ASHA, 2022; Cannon & Guardino, 2022; Speech Pathology Australia, 2016). EI approaches are traditionally based on a European-American cultural perspective and can contain implicit cultural biases related to the values and beliefs that underlie parent–child interactions (Puig, 2010; Van Kleeck, 1994). The risk of implicit cultural bias is highlighted by a recent review which showed that less than a quarter of interventions for CLD children in the past four decades are linguistically *and* culturally responsive (Larson et al., 2020).

Culturally competent services are required for all CLD families, including children with hearing loss. Cultural competence refers to the ability to respect beliefs, languages, and behaviors of the families, as well as of other professionals (Betancourt, Green, Carrillo, & Ananeh-Firempong, 2003). Culturally competent services should provide CLD families with the same quality and quantity of information given to families from the majority culture, and services should be provided in a way that demonstrates respect for cultural differences (Verdon, Wong, & McLeod, 2016; Yoshinaga-Itano, 2014). Cultural competence also includes approaching CLD families without any assumptions about their behaviors or perspectives, but knowing the topics where families may have attitudes, beliefs, and patterns of engagement that differ from those of the community (Bergeron & Beauregard, 2018; Leigh & Crowe, 2015; Stewart & Applequist, 2019).

Second, for parents to create optimal communicative environment in their child’s everyday life, they need to hold relevant knowledge and believe in their ability to effect changes in their life and the life of their children (Alper et al., 2021; De Houwer, 2017; Leffel & Suskind, 2013). For parents of children with hearing loss, creating of an optimal communication environment requires professionals to understand the family’s beliefs, assumptions, biases, and knowledge on a range of topics, including the cause of the hearing loss, beliefs about signed languages, and expectations for children with hearing loss. All parents’ engagement in partnerships, such as those required for FCEI, depends on daily constraints (administrative or financial situation, social support), personal issues (mental health, interpersonal skills) and—in the case of CLD parents—factors related to the CLD status (skills in the societal language, understanding of the health care system) (Brassart, Prévost, Bétrisey, Lemieux, & Desmarais, 2017; Staudt, 2007).

Finally, between professionals and parents, cultural differences can lead to divergent beliefs, expectations, and approaches to family involvement, which can negatively affect the partnerships (Verdon et al., 2016). In FCEI, it is therefore recommended that parents and professionals discuss and adjust their expectations, and identify how to practice, while respecting their views (Verdon et al., 2016). As EI focuses on counselling and support, linguistic differences between professionals and parents can affect communication, and limit the amount of information and resources available for the families (Bowen, 2016). Some resources may be needed to bridge the gap such as using interpreters, translations, written materials, visual cues, or repeated explanations from the providers (Grandpierre et al., 2019).

## Clinical Issues with CLD Families

In clinical practice, professionals often encounter difficulties in working with CLD families. This is exemplified by the findings of Crowe, McLeod, and Ching (2012), who compared the rates of multilingualism of parents and their three-year-old children with hearing loss in a population-based study in Australia. They reported that while 19.9% of female caregivers and 21.1% of male caregivers were multilingual, only 12.7% of their children were multilingual in their home environment and only 2.1% of their children received multilingual early intervention services. There are many reported sources of difficulties for professionals providing multilingual services. These include a lack of information about multilingual speech and language acquisition (D’Souza, Bird, & Deacon, 2012; Guiberson & Atkins, 2012), lack of information on culturally responsive interventions (Cycyk et al., 2021), difficulties to find appropriate information or clinical resources to support multilingual children and their families (Crowe & Guiberson, 2021), barriers related to linguistic or cultural differences (Grandpierre et al., 2019), and reduced collaboration with families (Bijleveld et al., 2014; Williams & McLeod, 2012). Despite recommendations from professional associations and calls in the literature to include more multilingual content in the training for SLPs (Williams & McLeod, 2012), professionals such as SLPs continue to report they feel unprepared to work with CLD families (Bijleveld et al., 2014; Caesar, 2013; Newbury et al., 2020; Williams & McLeod, 2012). Therefore, the increasing cultural and linguistic diversity among young DMLs poses specific challenges in EI and traditional perspectives and practices appear to be inadequate to meet these new challenges.

## The Current Study and Research Aims

The current study takes place in the highly CLD city of Brussels, capital of Belgium and center of the European Union, where over 100 different languages are reported to be spoken in the city (Extra & Yağmur, 2011). This study aims to build a better understanding of the situation by providing a preliminary exploration of the perspectives and practices of two key stakeholders in FCEI for DMLs—parents and professionals—in a linguistically diverse non-English context. The aim of this study was to understand the specific role SLPs play in EI for families with DMLs, and to examine the needs that SLPs and parents of DMLs consider are necessarily to improve EI services for DMLs and their families.

## Method

### Participants

#### Speech-language pathologists

Thirteen Belgian French-speaking SLPs who provided EI services to children with hearing loss aged 0–3 years and their families participated in this study. The majority worked in rehabilitation centers specifically serving children with hearing loss (*n* = 11, 84.6%) with the others working in private practice (*n* = 2, 15.4%). SLPs generally provided sessions for the children once (*n* = 3, 23.1%) or twice (*n* = 10, 76.9%) a week and met with parents once (*n* = 6, 46.1%) or twice (*n* = 7, 53.9%) a week. Participants had between 4 and 40 years (*M* = 20.2, *SD* = 11) of clinical experience as SLPs.

#### Parents

Five mothers of DMLs participated in an interview for this study. Characteristics of the mothers are presented in Table 1. At the time of the interview, the children were aged between 34 and 58 months (*M* = 46.4, *SD* = 9.2). Their children were all born in Belgium and had all received SLP services that involved parental support in Belgium within the first three years of their lives. The age at intervention began was between 5 and 12 months (*M* = 8.6, *SD* = 3.3).

**Table 1:** Cultural and linguistic characteristics of the mothers

	Country of origin (country of birth)	Home language(s)	Shared language with the child’s SLP
Mother 1	Morocco (Belgium)	Moroccan Arabic, French	French
Mother 2	Burkina Faso	Mooré, French	French
Mother 3	Turkey (Bulgaria)	Turkish	None
Mother 4	Poland	Polish	English
Mother 5	Morocco	Moroccan Arabic, French	French

### Procedure

#### Recruitment

*Speech-Language-Pathologists.* Information about the study was sent to SLPs working in early education centers specializing in the support of children with hearing loss in Brussels, Belgium. The information contained a link to an online survey made with Google Forms. To participate, SLPs had to be currently working with children with hearing loss aged 0–3 years.

*Parents.* Staff at a center for children with hearing loss in Brussels, invited families to participate in this study based on the following criteria. That families had: (a) linguistically diverse backgrounds (i.e., at least one family language that was not French, Dutch or German, which are the official languages of Belgium), (b) children currently aged 4–5 years, and (c) received services from that center when their child was aged 0–3 years. Eligible families were approached by their regular social worker or SLP and parents were provided with information about the study. Written consent was collected from parents who wished to participate in this study. None of the SLPs who were working with the children aged 4–5 years had worked with those children earlier when they were 0–3 years old. This ensured that parents would not be asked to report on their experiences with the professionals who were currently providing them with intervention services.

#### Data collection

##### Speech-Language-Pathologists

SLPs completed an online survey that was custom designed for this study. As this was a small-scale and preliminary study of this phenomena in Belgium, the questionnaire was designed by the first author based on her clinical experience and observations working with DMLs in Belgium. The questionnaire was piloted with two French-speaking Belgian SLPs who examined whether questions could be clearly understood, if all questions were relevant, and if any questions they felt were necessary were missing. Adjustments were made to the questionnaire based on their feedback.

In the questionnaire SLPs were asked to share their experiences of providing SLP services to *parents of foreign origin who use at least one language other than French at home with a deaf child aged 0–3 years*. It was explained that this included parents who can speak French in addition to their home language, and those who do not speak French. This is a typical way of describing families of DMLs in Belgium. The questionnaire containing six closed and eight open-ended questions. Closed questions gathered demographic information about the participants and their professional experience. Questions also elicited information about the frequency of SLP sessions with the families of DMLs (once a week, twice a week, once or twice a month, less frequently than this, other), self-satisfaction with service provision (0 = not satisfied at all, 10 = completely satisfied), and feelings of mastery of professional skills (total lacking, rather lacking, some mastery, complete mastery). These ratings were gathered for participants’ interactions with families of DMLs and families of children with hearing loss who were not CLD (mainstream families). Open-ended questions asked SLPs to describe the adaptations they made in their usual practice for clients who were multilingual and from different cultures, the difficulties and challenges faced in supporting parents of DMLs, and their professional needs in responding to diversity.

##### Parents

Parents were asked if they would prefer to complete the interview in French or in another language with an interpreter. Three parents completed the interview in French and two completed the interview with an interpreter. Interviews were an approximately 30 minutes in length and were conducted either at the EI center or at the family’s home, depending on the preference of the parent. The interview consisted of six closed questions about demographic information and five open-ended questions about parents’ perspectives on the support they received from SLP in EI (e.g., Did you feel that your child’s multilingualism was taken into consideration in early intervention?). The open-ended questions required relatively short answers. Parents’ responses were transcribed during the interviews.

#### Data analysis

A mixed methods approach was used to analyze data in this study. Quantitative methods were used for the SLPs’ responses to binary and Likert-style questions in the survey. Descriptive and inferential statistics were conducted in JASP software (JASP Team, 2019). Given the small sample size and non-normal distribution of the data, differences were examined using the nonparametric Wilcoxon test. Inductive qualitative analysis (based on the methods of Braun & Clarke, 2006) was used to qualitatively examine SLPs’ responses to open-ended survey questions and parents’ interview responses. The analysis was conducted by two researchers (the first author and a research assistant). First, data were read through several times by the two researchers. During reading, they made annotations about comments or ideas that occurred and that could be relevant for the coding step. Second, initial codes were generated independently by the two researchers. Coding took place in a dynamic way, allowing for changes in the content and structure of coding. Third, the two researchers compared their codes and discussed these until consensus was reached. Fourth, the first author independently generated themes and sub-themes from the codes and made a thematic map to illustrate how the themes fitted together. Fifth, the other researcher gave feedback on codes, sub-themes, and themes and data interpretation. Sixth, modifications were made to codes, sub-themes, and themes were these were then applied consistently to all data by the first author. Finally, themes, sub-themes and codes were tabulated (see Table 2).

**Table 2:** Themes, sub-themes, and codes identified in the data

Theme	Sub-theme	Codes	Responses		
			Total mentions	Number of SLPs (*n* = 13)	Number of mothers (*n* = 5)
Communication and partnership	Support for oral communication	• Images, video	15	8	0
		• Multilingual documentation	11	6	1
		• non-verbal, gestures, signs	8	7	0
		• Simplified spoken message	6	5	0
		• Interpretation and translation	6	4	1
	Necessity of effective communication	• Need for shared language	19	12	1
		• Precisions and nuances	8	6	0
		• Communicative efforts	7	6	0
	Multilingualism of the child	• Discussing language use	12	5	5
		• Informing parents	9	5	3
		• Monitoring development infamily language	5	3	0
	Type/nature of parental support	• Negotiation of the partnership	8	5	1
		• Request of parents	2	2	0
Professional resources for responding to diversity	Practices	• Implementation in real-timeinteractions with the child	9	6	0
		• Examples and modeling	7	4	0
		• Adjust to individualcircumstances	7	5	2
		• Multidisciplinary team	2	2	0
	Knowledge	• Languages and cultures	6	4	0
		• Multilingualism	5	3	0
	Attitudes	• Respect	8	4	4
		• Sensitivity	4	3	0
Diversity of parental profiles	Personal characteristics	• Beliefs	10	5	0
		• Socioeconomic status	4	2	0
		• Capacity to talk aboutlanguage	4	3	0
		• Emotions	4	4	0
		• Acculturation	4	2	1
		• Expectations	3	2	0
	Parenting practices	• Child-rearing	8	6	0
		• Interactive style	3	3	0

### Situating the Researchers

The data collection and analysis in this study was conducted by the first author and a research assistant. As qualitative research is inherently influenced by the subjectivity of the researchers, it is necessary to understand the background of the researchers and how this may have influenced the viewpoints, which have been brought to the analysis. The first author is a Belgian SLP who uses French, Belgian Sign Language, and English. She has experience working with DMLs and children with speech and language disorders in Belgium and Canada. She is also in charge of learning activities about multilingualism for SLP master’s students. As the SLP community in Belgium is small and few SLPs work with children with hearing loss, some of the participants were known to the first author. The research assistant is a Belgian French-speaking SLP. She has experience working with children who have language disorders associated with autism spectrum disorder, Down syndrome and motor disorders in Belgium and Canada. She has experience in conducting qualitative thematic analysis.

## Results

### Professionals’ Perspectives

**Figure 1:**
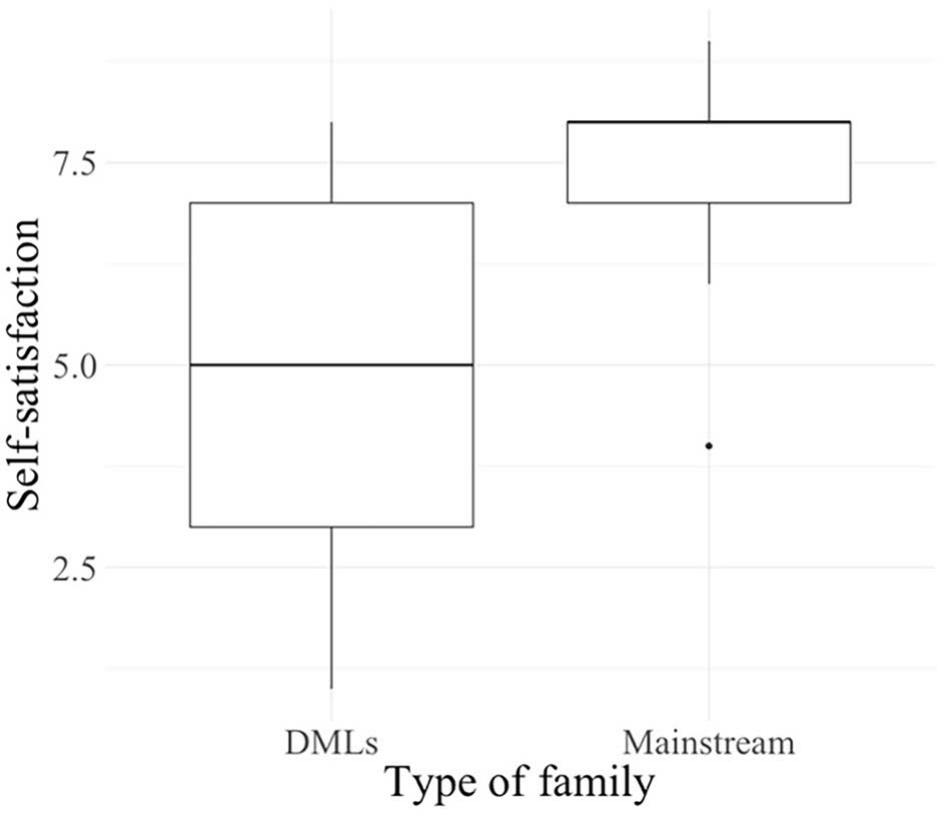
SLPs self-satisfaction in supporting families (maximum score 10).

**Figure 2:**
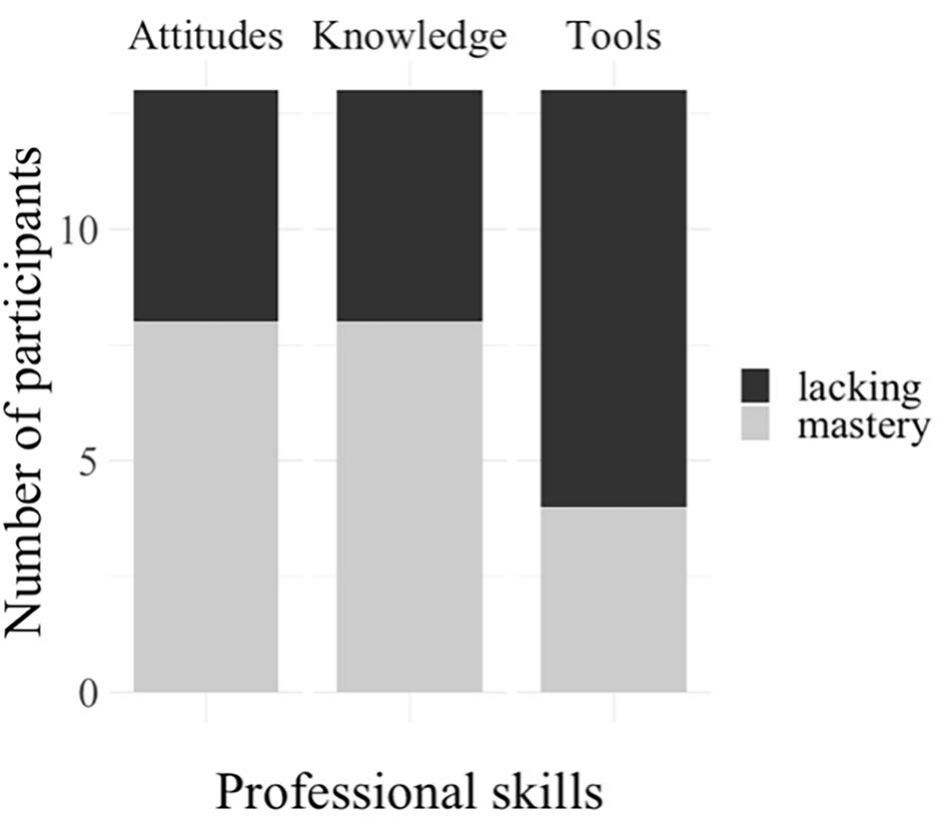
SLPs feeling about skills to support families of DMLs.

SLPs were asked to rate their self-satisfaction in supporting mainstream Belgian families (non-CLD families of children with hearing loss) and families of DMLs on a scale of 0 (Not satisfied at all) and 10 (Completely satisfied). As shown in Figure 1, SLPs reported significantly higher levels of self-satisfaction in supporting mainstream Belgian families (*M* = 7.4, *SD* = 1.3) than families of DMLs (*M* = 4.8, *SD* = 2.0), (W = 91, *p* = .001). SLPs also rated their perception of their professional skills required to support families of DMLs (mastery/lacking) in three areas: knowledge, attitudes, and tools and strategies. The results showed that while 61.5% (*n* = 8) SLPs reported a mastery for both knowledge and attitudes, 69.2% (*n* = 9) reported a lack of tools and strategies (see Figure 2). The two SLPs with less than five years of experience (*n* = 2) reported their skills in all areas were lacking. Beyond five years of clinical experience, there was no clear relationship between years of experience and feelings of mastery.

### Parents and SLPs’ Perspectives

**Figure 3:**
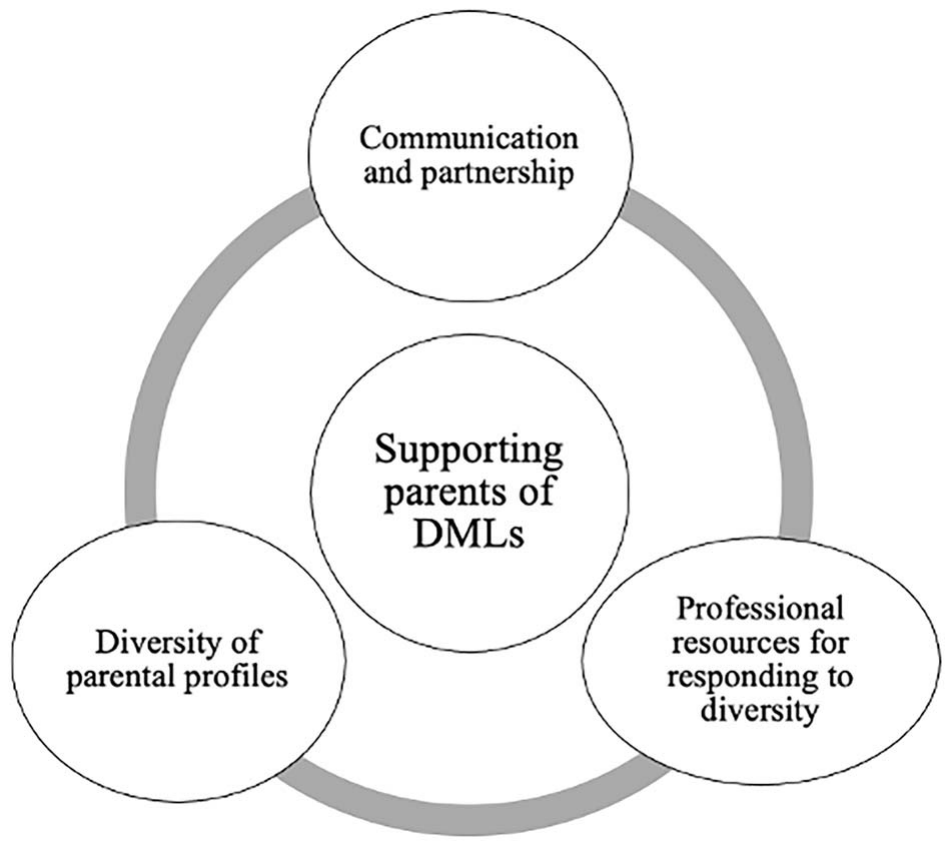
Thematic map of qualitative data.

Inductive thematic analysis was conducted for data from parent interviews and responses to open-ended questions in the SLP questionnaire. Three themes emerged: *communication and partnership, professional resources for responding to diversity,* and *diversity of parental profiles*. Themes, subthemes, and codes are presented in Table 2 and a thematic map of these themes is provided in Figure 3.

#### Communication and partnership

The theme *communication and partnership* described the adaptations needed in the communication between SLPs and parents of DMLs to understand each other despite linguistic and/or cultural gaps, to discuss issues related to the multilingualism of the child, and to determine the type of parental support to be provided. Four subthemes were identified: support for oral communication, necessity of effective communication, multilingualism of the child, type of parental support.

##### Support for oral communication

Participants reported their current practices and their needs in supporting oral communication between SLPs and parents. The codes illustrate different solutions for bridging linguistic gaps. SLPs described their use of illustrated documentation to overcome language barriers. One SLP explained: “I try to involve parents as much as possible by using visual supports to help them understand” (SLP9). The need for more illustrated documents and video clips on a range of themes (e.g., child language development, communication strategies) in French and other languages was also described. For example, visual supports demonstrating parent–child interactions and targeted behaviors: “For my part, I need to use video more as a tool ... to interact with parents, to show them typical interaction situations in their language and then ask them to reproduce them in front of me” (SLP3).

SLPs stated that they tried to find information in the family’s preferred language but that they need more multilingual documents in order to work effectively (e.g., information, advice, questionnaires). One SLP commented: “[We need] translated document to be completed by the parents to find out their expectations, practices with the child, beliefs about multilingualism, daily activities” (SLP1). Another suggested: “Perhaps a leaflet with the main developmental stages in several languages” (SLP4). One parent requested more written information, regardless of the language: “[I lacked] written material. Even in French, I could have asked other people to translate it for me, as I had very little communication with the SLP” (P17). Oral communication was supported by SLPs use of non-verbals communication, gestures, and signs: “I accentuate a lot with non-verbals, I show by pointing, I make natural gestures, sometimes even signs” (SLP2). SLPs also adapted their communication to better interact with families of DMLs: “I address [the parents] in simplified language, to make sure I am understood” (SLP12). Specific strategies mentioned by SLPs were using shorter sentences, simpler vocabulary, rephrasing, and using translation software. For specific meetings with parents, SLPs reported using professional interpreters: “I use an interpreter to present the assessment findings and initiate the intervention, but this is insufficient to explain the whole intervention process” (SLP1). Parents also commented on their preferences for interpreter use. For example, one parent said: “It would be good to have an interpreter during SLP sessions, like during this interview. It would help me to understand the purpose of what is being done. It wouldn’t be useful every time, but perhaps the first time to explain the work and goals of the SLP, and then at a debrief meeting a few months later” (P17).

##### Necessity for effective communication

Effective communication in EI sessions was mentioned as being necessary by both parents and SLPs. The lack of a common language makes effective communication challenging and, in some cases, impossible. Even with a shared language, one party may have a lower level of competence in that language, which means that precision and nuances in communication may be lacking. As a consequence, SLPs reported that greater communicative effort was required.

A shared language was described as a basis for many interactions between SLPs and parents, including considering parents’ questions, expectations, and feelings. The importance of a shared language in EI was mentioned by many participants (*n* = 13, 72.2%). One SLP stated: “If there is no common language, then referral is necessary: early help services, translators ... you can’t do it alone if there is no common language” (SLP13). Professionals reported needing more time to support parents of DMLs as communicating took longer and was more difficult: “It’s hard!” (SLP1) and “When both sides have to make efforts to understand each other, part of the natural dynamic of communication is already broken” (SLP3). Both parents and SLPs described that even if parents were competent (but nonnative) speakers of French, it was still hard to communicate in a precise way: “The language barrier is a problem to using nuance and giving more examples” (SLP11) and “To organize appointments, my French was enough, but the speech therapist’s work is too technical. I couldn’t understand” (P17).

##### Multilingualism of the child

Participants described the important place of the child’s multilingualism in EI, and in the discussions between parents and SLPs. SLPs and parents described discussions about which language to use with the child. All SLPs recommended that parents use their mother tongue with the child. One SLP elaborated on this point: “I insist on the development of the mastery of a basic language on which the other languages are grafted, and on the correct and rich practice of the mother tongue [by parents]” (SLP4). One parent said: “[The SLP] gave us a lot of encouragement to develop the family language with our child” (P17). One exception to this recommendation was for one multilingual family who chose to use French first and waited for the signal from the SLP to being using their other language with the child. SLPs also described providing parents with up-to-date evidence-based information on multilingual speech and language development and discussed with parents the advantages and disadvantages of multilingualism for the child. One SLP said: “I present the knowledge I have on the subject, based on current literature or conferences I have attended” (SLP3). This was confirmed by a parent who said: “The SLP explained to me that my child might have a delay but that it wouldn’t be due to multilingualism” (P15). SLPs collaborated with parents to monitor the child’s development of the family language. SLPs could monitor the development of French by direct observation or assessment. Parents were invaluable partners with SLPs in identifying the child’s skills in the family language. For example: “I discuss with the parent the [child’s] level of understanding and production in the family language” (SLP6).

##### Types of parental support

The type of support SLPs provide, and the degree of parental involvement, was described in many ways. While the partnership must be negotiated, parents’ requests for support were an important factor in the process of determining the type of parental support an SLP would provide. When SLPs and parents discuss to share their expectations, identify common goals, and agree on the type of parental support that will be implemented, they may experience some challenges. Some participants attributed this challenge to cultural and/or linguistic differences between the SLP and the parent. For example, one SLP said: “The weight of tradition and custom does not meet the urgency of the specific needs related to hearing loss” (SLP7). Another said: “[SLPs] need professional training on how to negotiate and co-construct a project with parents” (SLP5). Parents also commented on the nature of their partnership with SLPs: “It should be explained more clearly that the SLP is also there for the parents, not only for the child” (P15).

SLPs described how they adapted their support depending on parents’ requests. Some SLPs saw parental requests that deviate from what they would normally expect as a challenge: “Generally, these families do not ask for parental support. It is *imposed* because of the diagnosis of hearing loss and the SLP intervention. This can be experienced as an intrusion into ... their daily lives, into their habits” (SLP3). For other SLPs, the more active/passive profile of the parents was just a factor to consider in adapting their support: “I adapt the way I explain things according to several parameters such … [the parent’s] passivity or, on the contrary, their questioning” (SLP4).

#### Professional resources for responding to diversity

The theme *professional resources for responding to diversity* described the different resources specifically needed to work as a SLP with families of DMLs. Three subthemes were identified: practices, knowledge, and attitudes.

##### Practices

Support and coaching practices needed to be adapted when working with families of DMLs. Participants described concrete adaptations as well as the need for tools to compensate for linguistic gaps, tools to guide SLPs in how to adjust to individual circumstances such as cultural habits, and the need to be part of a multidisciplinary team. Parent coaching mainly occurred during real-time interactions between the SLP, the child, and the parent: “We play together, and everyone gives comments in their mother tongue. We triangulate and the interactions are fun” (SLP3). For one parent, the environment in which interactions with the child took place was important: “Thanks to the sessions at home, I knew how to communicate with my child” (P16). Coaching also occurred by modelling when oral communication was difficult or not effective: “I coach more, if not exclusively, through modeling and showing what I do with the child” (SLP1). SLPs also described the need for tools to help parents in learning new behaviors: “[we need] ideas, materials, or links to demonstrate [to parents]” (SLP5) and “[we need] a procedure for the therapist to model a behavior visually” (SLP1).

SLPs described that supporting families of DMLs required great adaptability: “About cultural differences, by learning how the family works, we can try to reach out to these families and offer them things that are relevant to them” (SLP6). However, this adjustment was difficult when SLPs couldn’t communicate effectively with the family about their preferences: “We have little or no possibility of knowing ... their daily wishes to work on spoken language. For example, they don’t read books and don’t see the point [of reading books]. How can we best adjust to this without imposing activities on them?” (SLP1).When SLPs were working with families with complex needs (e.g., low income, irregular status, health issues), being part of a multidisciplinary team was seen as an asset: “I work in a multidisciplinary team with therapists of different professions and sensitivities, and I can refer to one or the other depending on the situation, for the good of the client” (SLP13).

##### Knowledge

Specific knowledge on foreign languages, foreign cultures, and multilingual development was viewed as important by SLPs working with DMLs and their families. SLPs described their need to learn about other languages and about language acquisition in other languages: “[we need] phonetic charts in different languages, and the ages of acquisition of morphosyntactic structures in these languages” (SLP2). SLPs also highlighted their lack of knowledge about other cultures. For example: “I adapt very little to the culture because I don’t know the culture of the other person” (SLP7). Another SLP, with 25 years of clinical experience, added a different perspective: “Thankfully, practice helps over the years to get to know some cultures better” (SLP10). SLPs expressed both their use of and need for scientific information on multilingualism, multilingual development, and assessment of multilingual children. They need this information to inform parents but also to correctly analyze children’s speech and language development: “I sometimes discuss the child’s multilingual development with the parents, but I don’t feel comfortable enough with these theoretical notions” (SLP3) and “I need a better understanding of the implications of multilingualism for deaf children” (SLP8).

##### Attitudes

The interpersonal attitudes of both parents and SLPs can impact on how EI is engaged in. This is especially important for SLPs working with parents of DMLs. SLPs described their respectful and non-judgmental attitude towards family’s customs and parenting practices: “I don’t go against their educational principles. I listen to them, and I propose my own [ideas], ideally trying to find adaptations that would suit them best” (SLP3). Parents also reported respectful attitudes from the SLPs: “She didn’t make remarks or judgements about the way we did things as parents, but she adapted to it” (P16). However, while SLPs said that they respected family culture, most also said they did not really adapt their practices: “[with every family] the support itself remains the same” (SLP2), a point picked up on by a parent: “I think we got normal advice, like other families, regardless of culture” (P17). However, SLPs reported having a more sensitive attitude with parents of DMLs than with mainstream parents. As CLD parents may have parenting practices and interaction patterns that differ from mainstream families or from the SLPs’ cultural background, SLPs must learn about the practices of each family. Due to possible language barriers, SLPs reported using observation, listening, and their feelings rather than discussion to learn about each family’s habits: “[I take] more time to observe the interactions and practices with the child” (SLP7) and “I adapt according to what I feel about my interlocutor. Are they ready to receive this or that? Now or later?” (SLP3).

#### Diversity of parental profiles

The theme *diversity of parental profiles* described how parents differ from one another. Two subthemes were identified: personal characteristics and parenting practices.

*Personal characteristics* Several personal characteristics of parents were mentioned by SLPs as factors that impact on parental involvement and that need to be considered when adapting support. SLPs described the cultural influence on parental beliefs about child development, child-rearing, deafness, disability, and the role of SLP. For example: “The difficulty is, in my opinion, due to the gap between our educational beliefs from one culture to another” (SLP3) and “The belief of the practitioner to whom [parents] entrust their child with closed eyes without getting involved” (SLP4). The frequent co-occurrence of cultural and/or linguistic diversity and low socioeconomic status was another influencing factor. One SLP stated: “Multilingualism is not the problem, it is the socio-economic level, among immigrant parents” (SLP5).

In EI, the metacommunication skills are required by parents, which is the ability to think and talk about language and communication. This was seen as a challenge when working with families of DMLs: “[Difficulties with some parents are due to] their difficulty in understanding interactions, preverbal skills, and how to talk to the child” (SLP12). SLPs also described the changing nature of parental emotions related to their child’s hearing loss diagnosis, and the importance of taking this into account in EI. One SLP stated: “I adapt my way of explaining things according to several parameters such as … how the child’s disability is experienced and how long ago the diagnosis was, where the parents are in their process” (SLP4). Acculturation was also mentioned. While SLPs mentioned these influences as a challenge, parents mentioned EI as a positive opportunity for their own acculturation. For example, an SLP said: “[For some parents], life in Belgium is not a choice, the link with another culture is not voluntary, there is an ambivalence” (SLP7). A parent said: “The intervention with a Belgian SLP also helped me to integrate [in Belgium]” (P18).

*Parenting practices* Parents’ child-rearing practices and how they interact with their child may vary. These practices were considered by SLPs to adapt their support for families of DMLs. SLPs described the cultural influence on child-rearing practices and the possible gap between the family’s practices and the SLP’s advice. One SLP commented: “The (…) difficulty is, in my opinion, due to the gap between our educational beliefs from one culture to another, advocating autonomy or doing everything for the child, singing songs or never singing, explaining everything to the child or telling him nothing because he is too small to understand” (SLP3). Another said: “I try to adapt to the culture of the family, to know how they do with babies in the country of origin” (SLP5). Cultural and personal differences in the interactive style of the parents when talking to their children were also described. One SLP stated: “I am attentive and vigilant about the way [the parents] function and educate their child. I try to know if and how they talk to their child” (SLP6).

## Discussion

This study aimed to investigate the specific role of SLPs in EI with families DMLs, and to examine the needs of both SLPs and of parents in order to improve EI services.

Quantitative data identified clear patterns in SLPs’ perspectives of their professional practices and skills. SLPs expressed significantly lower self-satisfaction in supporting families of DMLs compared to mainstream families. Most of the SLPs felt comfortable with their professional skills in terms of their knowledge and their attitudes, however, most SLPs felt uncomfortable with the tools and strategies available for working with DMLs and their families. These results confirm the presence of difficulties in providing appropriate EI services to families of DMLs. This is consistent with previous surveys in which SLPs from other countries report difficulties in the collaboration with CLD families in general (Bijleveld et al., 2014; Williams & McLeod, 2012). To analyze the needs of SLPs and parents, as well as the factors involved, qualitative data allowed detailed examination of both SLPs’ and parents’ perspectives in EI settings. The discussion of results is organized around three main themes: the communication paradox, professional issues, and multifaceted diversity and FCEI.

### The Communication Paradox

This study illustrates a specific paradox regarding communication with parents of DMLs in EI. On the one hand, effective communication is needed to negotiate the partnership, to support parents in implementing appropriate strategies in their language, and to discuss the child’s multilingualism. On the other hand, linguistic differences between SLPs and parents increase the risk of miscommunications. Without a shared language between SLPs and parents, interactions required more communicative effort, SLPs gave less precise information and advice to parents, and miscommunications occurred easily. To overcome the language barrier, professionals required specific tools such as illustrated materials, video clips, or multilingual documentation, not all of which exist. Both SLPs and parents emphasize the relevance of using professional interpreters to bridge the language gap at key points in EI. These specific aspects of intervention with families of multilingual children converge with those from previous studies in different contexts (Bowen, 2016; Crowe & Guiberson, 2021; Grandpierre, Fitzpatrick, Thomas, Mendonca, et al., 2019; Grandpierre, Fitzpatrick, Thomas, Sikora, et al., 2019; Leigh & Crowe, 2015; Stewart & Applequist, 2019; Verdon et al., 2016). Findings in this study also highlighted pragmatic issues such as the need for additional time, resources, and funding for professionals working with families of multilingual children, as stated in the International Expert Panel on Multilingual Children Speech position paper (IEPMCS, 2012).

### Professional Issues

One specific aspect of supporting families of DMLs is the necessity to address questions around multilingualism early and frequently with families (Crowe, Fordham, McLeod, & Ching, 2014; Crowe & Guiberson, 2021). In this study, SLPs and parents reported frequent discussions focused on information and advice on language use. Most of the SLPs in the present study were confident in their knowledge about multilingualism, contrasting findings by D’Souza et al. (2012) and Guiberson and Atkins (2012) who reported a lack of information about multilingualism among Canadian and American SLPs. This difference may be due to the fact that SLPs in our sample were interested in—and therefore probably more comfortable with—issues related to multilingualism. This could also be explained by differences in the initial education of SLPs between Belgium and North America, by evolution in professional education over the past decade, or by the different caseload demands of SLPs practicing in these two contexts. If SLPs and parents reported discussing language use, it is mainly SLPs recommending parents use their first language with the child. This recommendation confirms an evolution in the knowledge of professionals since the early 2000’s (Crowe & McLeod, 2016) or shows a context that has not been previously investigated for professionals working with children with hearing loss. However, the process of enabling parents to make an informed choice about language use and communication mode goes beyond providing this initial recommendation. Informed choice requires professionals to provide relevant information about the full range of options available and, because providing information alone does not guarantee understanding, to empower parents to be active decision makers (Carr et al., 2006). In this study, it is possible that while the SLPs support parents with some aspects of making informed choices about language use, they don’t engage completely with parents in this process.

In the thematic analysis of data collected in this study, SLPs indicated the need for information on different languages and cultures, and information on multilingualism in DMLs. Knowledge about these topics is specifically required when working with multilingual children, and some resources are already available, such as the Intelligibility in Context Scale (McLeod, Harrison, & McCormack, 2012) and phonetic description of languages (McLeod, 2012). However, the majority of resources are in English, and they may not be easily accessible to SLPs who speak and practice in other languages. The same is true for research evidence on multilingualism for children with communication disorders, and DMLs in particular. In previous research, professionals have reported not having the time or access to keep up to date with relevant scientific literature (Crowe & Guiberson, 2021) and this difficulty may be compounded for professionals whose preferred language is not English. Even if SLPs in this study were confident in their knowledge, the majority reported a lack of tools and strategies to support families of DMLs. This finding was consistent with previous research reporting that professionals had difficulties in finding appropriate resources to work with DMLs and their families (Crowe & Guiberson, 2021; Newbury et al., 2020; Williams & McLeod, 2012). The universality of this issue is demonstrated in the IEPMCS (2012) position paper, which recommends: “SLPs generate and share knowledge, resources and evidence nationally and internationally to facilitate the understanding of cultural and linguistic diversity” (p. 2).

Most of the SLPs in the present study felt comfortable with their professional attitudes related to diversity. SLPs and parents mentioned a respectful and non-judgmental attitude towards cultural differences, but the results were more mixed regarding the adaptation to these differences. Some SLPs reported a more receptive attitude to learn and adapt to practices and preferences of the families of DMLs, compared to mainstream families. Other SLPs reported little adaptation, with support and advice remaining broadly the same for every family. SLPs pointed out the possible mismatch between the family routines and common advice or strategies in EI. Most of the time, SLPs seemed powerless to address this mismatch. This risk of mismatch was previously pointed out by Van Kleeck as early as 1994, due to implicit cultural biases that she identified in parent training programs, and this is unfortunately still relevant. In a recent review about adaptations of early language interventions for CLD children, it appears that most of the studies typically addressed children’s language (s) but not culture (Cycyk et al., 2021). The authors stated that SLPs were aware of the need to provide culturally and linguistically responsive EI but faced challenges to meet this directive. SLPs in Belgium may not be aware of the cultural bias present in professional practices or the need to provide culturally *and* linguistically responsive services.

The concept of cultural competence was not explicitly mentioned by the participants; however, in the current study some elements were related to this concept: the ability to respect the beliefs, language, and behaviors of the families (Betancourt et al., 2003), the provision of services in a way that demonstrates respect for cultural differences (Yoshinaga-Itano, 2014), and the need of knowledge about foreign cultures (Grandpierre, Fitzpatrick, Thomas, Mendonca, et al., 2019). Verdon (2015) proposes cultural competence as an active ongoing process of professional development that enables SLPs to provide services that are effective, useful and relevant to the needs of each family. The fact that cultural competence was not explicitly mentioned by participants in the present study is concerning, especially as working with families of DMLs was part of their daily practice. This may indicate that dissemination of information and materials related to cultural competence in Brussels, and beyond, would benefit the implementation of better services for families of DMLs. A shift to more culturally competent practices would also support SLP practices in areas, which they reported that a lack of tools to work with families of DMLs was problematic. This shift in perspective could best be described as using a *tailor-made* practice instead of waiting for a *ready-to-use* practice.

### Multifaceted Diversity and FCEI

The need to offer tailor-made services to families of DMLs is related to the diversity of parental profiles, one of the themes that emerged from the thematic analysis. In this study, because the sample included exclusively parents of DMLs (and no other disorders), heterogeneity among parents was limited. However, the parental profiles were still diverse and support the need for individualized services. SLPs reported diversity among parental beliefs and emotions related to hearing loss, and among beliefs on child language development, the role of SLP, and child-rearing. This is in line with the fact that, due to cultural and personal variations, parents may have beliefs, attitudes, and engagement patterns that differ from those of the mainstream community (Leigh & Crowe, 2015). SLPs also identified variations in parent characteristics other than multilingualism and hearing loss, but which are some related: family socioeconomic status, parental capacity to talk about language, and parental expectations for their child. These parental factors can all be addressed in interventions specifically tailored to parents from low socioeconomic level, whose children are at risk for language difficulties or who have hearing loss (Suskind et al., 2016). These findings show that cultural and linguistic diversity goes beyond cultures and languages and infiltrates many aspects of family life and professional practice. FCEI is particularly well suited to respond to this multifaceted diversity (Moeller et al., 2013; Voss & Stredler-Brown, 2017). A cultural shift is required from traditional models of service delivery—that were all child-centered—towards FCEI in order to improve services, as well as professional satisfaction, especially in highly culturally and linguistically diverse geographical contexts.

### Limitations and Future Research

This study has some limitations that provide opportunities for future research. As this was a preliminary study of this topic, the sample size was relatively small, included only SLPs working with children with hearing loss, and recruitment of SLPs may have been biased towards those with an interest in DMLs. As a result, the findings may not be representative of all SLPs working in EI in Belgium or in other EI settings. Future research should consider a larger sample, including SLPs from other French-speaking European regions, and other EI settings. Among the multiple stakeholders in EI, this study included only SLPs and mothers of DMLs. This may have led to a bias in the responses and a lack of alternative perspectives. Future research could include other professionals (e.g., ENT doctors, pediatricians, teachers in special education), parents of children with other disorders, and other family members (e.g., fathers, siblings, extended family members). Participants in the online survey gave some ambiguous responses that were difficult to interpret and resulted in lost data. Future research could include questionnaires with more accurate questions to avoid misunderstandings, or face-to-face interviews and focus groups to provide opportunity to seek clarification. As the survey and interview guide were based on looking for differences between the support of two types of families, this could have introduced a bias. Future research could reduce this bias in the formulation of the questions. Finally, the perspectives expressed in this study are subjective reports, and could be supplemented by direct observation of discussions between SLPs and parents, or coaching sessions.

### Clinical Implications

Families of DMLs come to EI services with individual characteristics and circumstances and need to be treated as unique. This study can help professionals become aware of the specific aspects of their role in EI with families of DMLs, and it can lead to self-reflection on professional skills that need to be developed. As a result of this work, clinical resources were developed and made easily accessible with other existing resources related to cultural and linguistic diversity for French-speaking professionals (see www.aloadiversity.com). Parent-friendly information on multilingual language acquisition that professionals can use were created and these resources are freely available and currently translated into eight languages (https://www.aloadiversite.com/infos-et-conseils-aux-parents). Responding to diversity in EI is a global issue. Graduate SLP programs could play a role by promoting cultural and linguistic diversity among their students and by including in their curricula more topics related to FCEI or cultural and linguistic diversity. This study shows that information and materials related to cultural competence need to be disseminated, both in graduate education and in continuing (self-) education, especially in highly CLD contexts. Part of the solution may also come from professionals facing this challenge in various regions, through the creation and sharing of resources. Local (or broader) professional associations could play a role in hosting or managing resource sharing systems. Finally, this study reiterates previous recommendations regarding health care policies and institutions. These include providing professionals with additional time, so that they can offer appropriate services to families of DMLs.

## Conclusion

This study confirms the specific role of SLP support for parents of DMLs in EI services, and the needs in current practice that should be addressed to improve EI services. The role of SLPs was to (1) develop partnership despite cultural and/or linguistic differences, (2) discuss multilingual development of the child and language use in the family, and (3) provide highly adaptable services due to the multifaceted diversity of families. SLPs showed positive attitudes towards multilingualism and reported many adaptations and efforts to implement effective intervention practices. However, they expressed low and variable self-satisfaction in supporting families of DMLs. To fulfill their role, SLPs need appropriate training (to develop cultural competence), knowledge (on multilingualism, languages and cultures) and resources (tools and strategies to overcome cultural and/or linguistic differences, additional time). In general, parents were very satisfied with the support they had received from the SLPs. They reported variability in the adaptation of SLP services to their language and culture, and difficulties to involve when there is no shared language. Some parents suggested that support could have been improved with interpreters or written documents. More research and evidence-based tools are needed so that SLPs can provide culturally and linguistically appropriate EI services that meet the needs of each family.

## Funding

Dr Daniël De Coninck Fund [Grant number 2019-J5170825-211765]; and the Belgian Kids Fund for Pediatric Research [Grant Antoine d’Ansembourg].

## Conflict of Interest

No conflict of interest was reported.
